# Phosphate-Solubilizing Bacteria Isolated from Phosphate Solid Sludge and Their Ability to Solubilize Three Inorganic Phosphate Forms: Calcium, Iron, and Aluminum Phosphates

**DOI:** 10.3390/microorganisms10050980

**Published:** 2022-05-07

**Authors:** Fatima Zahra Aliyat, Mohamed Maldani, Mohammed El Guilli, Laila Nassiri, Jamal Ibijbijen

**Affiliations:** 1 Environment and Valorization of Microbial and Plant Resources Unit, Faculty of Sciences, Moulay Ismail University, Meknes 50070, Morocco; nassiri_layla@yahoo.fr; 2 Department of Biological & Forensic Sciences, Fayetteville State University, 1200 Murchison Road, Fayetteville, NC 28301, USA; mohamed.maldani@gmail.com; 3 National Institute of Agricultural Research, Regional Center for Agricultural Research, Kenitra 14000, Morocco; mguilli@yahoo.com

**Keywords:** phosphorus, inorganic phosphate, phosphate solubilization bacteria, bioavailable phosphorus

## Abstract

Biofertilizers are a key component of organic agriculture. Bacterial biofertilizers enhance plant growth through a variety of mechanisms, including soil compound mobilization and phosphate solubilizing bacteria (PSB), which convert insoluble phosphorus to plant-available forms. This specificity of PSB allows them to be used as biofertilizers in order to increase P availability, which is an immobile element in the soil. The objective of our study is to assess the capacity of PSB strains isolated from phosphate solid sludge to solubilize three forms of inorganic phosphates: tricalcium phosphate (Ca_3_(PO_4_)_2_), aluminum phosphate (AlPO_4_), and iron phosphate (FePO_4_), in order to select efficient solubilization strains and use them as biofertilizers in any type of soil, either acidic or calcareous soil. Nine strains were selected and they were evaluated for their ability to dissolve phosphate in the National Botanical Research Institute’s Phosphate (NBRIP) medium with each form of phosphate (Ca_3_(PO_4_)_2_, AlPO_4_, and FePO_4_) as the sole source of phosphorus. The phosphate solubilizing activity was assessed by the vanadate-molybdate method. All the strains tested showed significantly (*p* ≤ 0.05) the ability to solubilize the three different forms of phosphates, with a variation between strains, and all strains solubilized Ca_3_(PO_4_)_2_ more than FePO_4_ and AlPO_4_.

## 1. Introduction

Phosphorus (P) is one of the major macronutrients for plant growth [1]. Therefore, in intensive agriculture, a supply of phosphorus in the form of fertilizer is crucial to obtaining good yields. As the reserves of degradable natural phosphorus are limited, a targeted and environmentally friendly supply is necessary for agriculture.

P is present in a range of organic and inorganic forms in soils. Inorganic P is typically found in soil as insoluble mineral compounds, some of which develop after continuous chemical fertilizer treatments [2,3]. However for plants, soil phosphorus is generally accumulated in chemical forms which are unavailable to them [4]. It usually takes the form of a phosphate ion, which is a charged ion that is bonded to other components to make a molecule. Such insoluble compounds comprise calcium phosphate in alkaline soils, and iron phosphate, and aluminum phosphate in acidic soils. Referring to Zou et al. [5], only ≈0.1% of the total P reserve in the soil is in the soluble form and available for plant uptake; generally, calcium phosphate is more soluble than aluminum phosphate, while the latter is more soluble than iron phosphate [6]. Most of the phosphates in the soil are absorbed into soil particles or incorporated into soil organic matter [7]. As well, a great portion of the soluble phosphate applied to the soil as chemical fertilizer is immobilized rapidly and becomes unavailable to plants [1,8].

Soil microorganisms like bacteria, fungus, and actinomycetes are involved in a variety of processes that lead to the transformation of soil P and are thus an integral part of the soil P cycle. In particular, soil microorganisms are efficient in releasing and unblocking inorganic and organic P from total soil P via solubilization and mineralization [9]. Currently, the primary goal of soil phosphorus management is to maximize crop yield while minimizing P loss from soils. The ability of a PSB to convert insoluble forms of phosphorus to accessible forms is an important trait in plant growth-promoting bacteria for increasing plant yields, and the use of PSB as inoculants to increase plant P uptake has increased [1,10]. 

In recent years, various studies have presented a large number of new PSB [10,11,12,13,14,15]. A great percentage of those were preliminary studies, conducted only in vitro and without plant and field application. Most of these studies assume that in vitro P solubilization ability will translate into available P for plant uptake in the soil.

To assess bacteria’s ability to dissolve insoluble phosphates, solid and liquid media techniques are used, and are based on media with a source of phosphorus unavailable to the bacteria [6]. The solid medium gives qualitative solubilization efficiency; the solubilization index is estimated by measuring the growing diameter of the colony and the solubilization halo in plates [16]. Moreover, the liquid medium gives quantitative solubilization efficiency through determining the pH changes and the soluble phosphate concentration in the medium [17]. Although most researchers up to now have relied on calcium phosphate as a universal source of phosphate to determine and judge that the bacteria tested are phosphate solubilizers, the authors of [8] recommended that the sole use of this phosphate to identify soil microorganisms as potential P solubilizers is not sufficient and that aluminum and iron phosphates should be tested as well. 

Phosphate solid sludge is a byproduct of the phosphate extraction industry’s exploitation and subsequent metallurgical treatment. The processing of phosphates creates a lot of sludge, which accumulates, forms fillings, reduces arable land, and changes the landscape. The phosphate sludge is mainly composed of phosphorus, minerals, and some of the original pollutants. To obtain some of these minerals, such as phosphorus, we thought about using this sludge as an agricultural substrate. One of the stages of this project was to isolate PSB from this sludge in order to valorize it as a biofertilizer. With the emphasis on isolation and screening of potential PSB for agricultural aims, this study was performed to evaluate the biochemical and genetic characteristics of PSB isolated from the phosphate solid sludge and to evaluate their capacity to solubilize three forms of phosphates unavailable to plants: calcium phosphate (Ca_3_(PO_4_)_2_), aluminum phosphate (AlPO_4_), and iron phosphate (FePO_4_).

## 2. Materials and Methods

### 2.1. Isolation and Screening of Phosphate Solubilizing Bacteria

Phosphate solid sludge samples were collected from the phosphate mining center of Khouribga (Morocco; 32°45′17.7645″, 006°51′14.5182″); One gram of each phosphate solid sludge sample was added to 9 mL of phosphate buffer saline (pH 7.2), serial dilutions from 10^−1^ up to 10^−6^ were realized. Then, 100 µL of 10^−3^ to 10^−6^ serially suspension was spread on NBRIP solid medium (10 g/L d-glucose, 5 g/L magnesium chloride hexahydrate, 0.25 g/L magnesium sulfate heptahydrate, 0.2 g/L potassium chloride, 0.1 g/L ammonium sulfate, amended with 5.0 g/L tricalcium phosphate (Ca_3_(PO_4_)_2_) as a sole source of P [18]. Then the Petri dishes were incubated at 30 °C for 5 days. The bacterial colonies with a clear halo zone were selected and purified three times on NBRIP solid medium. The qualitative efficiency of the selected PSBs was tested according to the solubilization index, measured by the formula PSI = C + H/C, (C = Colony diameter; H = Halo zone diameter) [16]. The cleaned isolates were kept on nutrient agar plates and stored at 4 °C, and a copy of each isolate was stored as a glycerol 40% stock at −30 °C.

### 2.2. Molecular Characterization of Selected PSB

The isolates showing the most pronounced P solubilizing activity (indicated by the solubilization index) were selected for further analysis. DNA extractions from these isolates were set, the extraction of DNA was achieved using the PureLink^®^ Genomic DNA Mini Kit (Invitrogen, Waltham, MA, USA, K1820-01) following the steps defined by the manufacturer, modified for Gram-negative bacteria. PCR amplification of the 16S rRNA of the bacterial strains was made using the DreamTaq PCR Master Mix (Invitrogen), containing of 22 mM Tris-(hydroxymethyl) aminomethane hydrochloride (pH 8.4), 55 mM potassium chloride, 1.65 mM magnesium chloride, 220 μM 2R,3S,5R)-5-(2-Amino-6-oxo-1,6-dihydro-9H-purin-9-yl)-3-hydroxyoxolan-2-yl]methyl, 220 μM 2R,3S,5R)-5-(6-aminopurin-9-yl)-3-hydroxyoxolan-2-yl]methoxy-hydroxyphosphoryl, 220 μM hydroxy-[[(2R,3S,5R)-3-hydroxy-5-(5-méthyl-2,4-dioxopyrimidin-1-yl)oxolan-2-yl]méthoxy]phosphoryl, 220 μM 2R,3S,5R)-5-(4-amino-2-oxopyrimidin-1-yl)-3-hydroxyoxolan-2-yl]méthoxy-hydroxyphosphoryl, and 22 U recombinant Taq DNA Polymerase/mL.

The universal primers 27F (f*orward*) (5′AGAGTTTGAT CCTGGCTCAG-3′) and 1492R (*reverse*) (5′-ACGGTTAC CTTGTTACGACTT-3′) were used to amplify a 1500 pb fragment, that corresponds to the genes of the bacterial 16S rRNA. PCR products were purified with the PureLinkTM Quick Gel Extraction & Purification combo kit (Invitrogen K220001), according to the manufacturer’s recommendations. Sequencing was made via primers 27F and 1492R, and carried out according to the Sanger technique adapted by the Big Dye Terminator V3 sequencing kit [19]. The ABI3730 DNA sequences permitted the automatic analysis of sequence reactions. The crude electropherograms were studied by MEGA7: Molecular Evolutionary Genetics Analysis version 7.0 for bigger datasets (Kumar, Stecher, and Tamura 2015), downloaded from www.megasoftware.net free of charge [19]. The consensus sequence from the f*orward* and *reverse* raw sequences were obtained for each strain; then, it was compared to other sequences using the BLAST server (blast.ncbi.nlm.nih.gov) to determine their phylogenetic affiliation [19]. The phylogenic tree was built via the neighbor-joining method [20].

### 2.3. Morphological, Biochemical Characterization

The selected strains were cultured on nutrient agar to study their morphological characterization, whereas for the observation of cell structure, Gram staining method was used. Biochemical characterization was carried out by the API 20 E system (API System, bioMerieux, Montalieu Vercie, France), following the manufacturer’s instructions. The API 20 E system is composed of 20 microtubes with dehydrated substrates inoculated with a bacterial suspension.

### 2.4. Plant Growth-Promoting Traits of PSB

The isolated strains were tested for their plant growth-promoting traits; for the Indole-3-Acetic Acid (IAA) production, the method described by Gordon and Weber [21] was approved; 200 µL of fresh bacterial cultures were inoculated in 30 mL of LB broth containing 0.1% l-tryptophan and incubated in the dark for 72 h in an incubator shaker at 28 °C and 140 rpm/min. The bacterial cultures were centrifuged at 10,000 rpm for 10 min. Then, 2 mL of supernatant was mixed with Salkowski reagent. After 30 min in dark, the optical density was measured at 530 nm using Ultraviolet and Visible Range Spectrophotometers UV-2005 (Spain). The quantity of IAA produced was determined by the standard graph of pure IAA. The siderophore production was determined on Chrome-Azurol S (CAS) medium following the Universal Chemical Assay according to the method defined by Schwyn and Neilands [22]. The development of yellow-orange halo around the cell was described as positive for siderophore production, and to detect the cyanide production by the strains selected, the method of Bakker and Schippers [23] was carried out. Plates observed for change in color of filter paper from yellow to orange to brown were described as positive for cyanide production.

### 2.5. Qualitative Analyses of Potassium (K) Solubilization

The following medium was used to assess the ability of isolate strains to solubilize potassium: 5 g d-glucose, 0.005 g magnesium sulphate heptahydrate, 0.1 g iron(III) chloride, 2.0 g calcium carbonate, 2.0 g calcium orthophosphates, 20 g agar, and 3.0 g mica as an insoluble K source per liter. The medium was autoclaved for 20 min to sterilize it. The medium was spiked with 0.25% bromothymol blue dye. The inoculated Petri plates were sealed and incubated for 72 h at 30 °C in an incubator [24]. Following the incubation period, the bacterial isolates’ ability to solubilize K was assessed qualitatively by looking for clear zones and a change in the color of the bromothymol blue dye from greenish blue to yellow.

### 2.6. Organic Acid Analysis by GC-MS

The PSB strains were grown in NBRIP broth medium for acid organic determination. Before autoclaving, 50 mL of NBRIP broth medium was adjusted to pH = 7.02 in a 250 mL flask. The medium was inoculated with 200 μL of fresh inoculum (1.8 × 10^8^ CFU/mL) and incubated in shaking conditions at 120 rpm/min at 30 °C for 72 h. 

Sample preparation: After centrifuging the sample for 5 to 10 min, 5 mL of supernatant was transferred to a Falcon tube. Considering the pH of the sample, 100 μL of sulfuric acid (1 N) was added. The pH was kept between 2 and 4. The samples were filtered in duplicate through a 0.2 µm filter and then, phenol (0.05 M) was added. 

Gas Chromatography coupled Mass Spectrometry (GC-MS) [25] was used to determine the presence of organic acids (acetic acid, formic acid, propionic acid, isobutyric acid, butyric acid, isovaleric acid, caproic acid, heptanoic acid) in the samples. The calibration curves of the standards were used to quantify the acids.

### 2.7. Inoculum Preparation 

The purified isolates were grown in the NBRIP medium. Each isolate was inoculated into test tubes containing 5 mL of NBRIP liquid medium and incubated for 24 h at 30 °C. Cells were harvested by centrifugation (Sigma 1-15K, Neustadt an der Weinstrasse, Germany) at 6400 rmp for 8 min, washed with 0.9% sterile saline, and were re-suspended to a 0.5 McFarland nephelometer standard to obtain an inoculum ~1.8 × 10^8^ CFU/mL [26].

### 2.8. Solubilization Test of the Three Forms of Phosphates

The solubilization test of the three forms of phosphates (Ca_3_(PO_4_)_2_, AlPO_4_, and FePO_4_) by the selected strains was estimated quantitatively and approved by using Erlenmeyer flasks of 100 mL containing 50 mL of liquid NBRIP medium adjusted to pH = 7.0 ± 0.2 before autoclaving amended with 5.0 g/L of calcium phosphate or aluminum phosphate or iron(III) phosphate as a sole source of P; then, inoculated with 200 μL of each isolate and incubated in shaking condition at 120 rpm/min at 30 °C for 7 days; 2 mL of the cultures were taken every 48 h and centrifuged at 10,000 rpm for 15 min. The content of soluble phosphate was estimated according to Murphy and Riley [27] using the molybdenum blue colorimetric method by measuring the absorbance at a wavelength of 882 nm with Ultraviolet and Visible Range Spectrophotometers UV-2005 (Spain). All treatments were in triplicate. The pH of the samples was also measured every 48 h with a digital pH meter.

### 2.9. Statistical Analysis

Data were analyzed using SPSS 20 software, and the results were expressed as the means ± standard deviation of three replicates. Data were examined by ANOVA, and post hoc mean comparison was performed by Duncan’s multiple range test at *p* ≤ 0.05. 

## 3. Results

### 3.1. Isolation and Screening of PSB Strains

From the phosphate solid sludge, 150 bacteria were recovered. On NBRIP media supplemented with Ca_3_(PO_4_)_2_ as the sole P source, all of the isolates were tested for phosphate solubilizing activity. Isolates were chosen based on their P solubility index (PSI). The isolates that produced haloes greater than 2 cm on plates were selected; 16% of the strains (BM11, BM28, CB13, CB19, BT125, BT3S171, BN313, BM218, and BM215) that demonstrated phosphate solubilizing activities on Petri dishes were chosen for further investigation (Table 1).

### 3.2. Morphological, Biochemical Characterization, and Molecular Characterization of Selected PBS

The selected PSB strains were all Gram-negative. BM11 colonies appeared shiny, red, and with round and smooth margins. All of the bacterial strains were yellowish, a little wet, transparent, and shiny. They were round but not smooth, and they all had the same shapes in morphology. Table 2 shows all the results of the biochemical test carried out.

**Figure 1:**
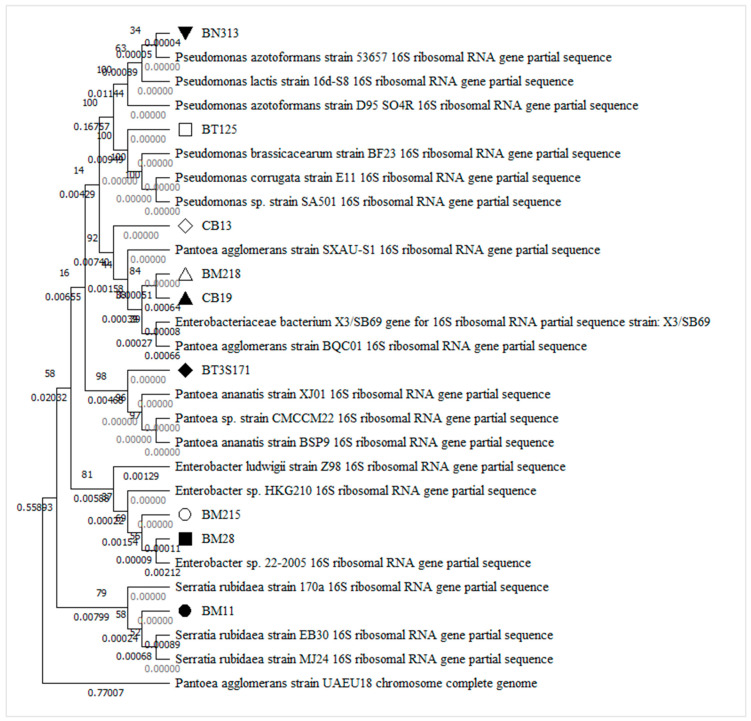
Phylogenetic tree built based on the partial 16S rRNA gene sequence using neighbor-joining method with Molecular Evolutionary Genetics Analysis version 7.0 for bigger datasets (Kumar, Stecher, and Tamura 2015), downloaded from www.megasoftware.net free of charge.

The molecular characterization of the nine PSB strains, based on 16S rRNA sequences, are presented in Figure 1. Four genera were identified: *Pseudomonas*, *Serratia*, *Pantoea*, and *Enterobacter*. Based on the analysis of the 16S rDNA partial sequence, the strain BM11 was identified as *Serratia rubidaea*. Strains BM28 and BM215 were identified as Enterobacter bugandensis. Moreover, the isolates CB19, CB13, and BM218 were identified as *Pantoea agglomerans*, and BT125 was identified as *Pseudomonas brassicacearum* supsp. *neoaurantiaca*, BT3S171 isolate was identified as *Pantoea stewartii* subsp. *Indologenes*., and strain BN313 was recognized as *Pseudomonas lactis*.

### 3.3. Plant Growth-Promoting Traits of PSB

The results of PSB’s Plant Growth-Promoting traits are shown in Table 1. Siderophore production was carried out on solid CAS blue agar. All tested isolates confirmed the secretion of siderophore with a significant difference between strains. BT125, BT3S171, BN313, BM218, and BM215 showed a high production of siderophore; all selected isolates demonstrated an important production of IAA with a significant difference between strains (p ≤ 0.05). Highest IAA production was reported in BT3S171 with 43.80 ± 0.39 μg/mL, followed by BM218 (20.53 ± 0.03 μg/mL) and BM215 (15.96 ± 0.20 μg/mL), and the lowest production of IAA was detected in BN313 with 1.37 ± 0.09 μg/mL. Out of the nine isolates, only BT125 isolate showed HCN production.

### 3.4. Qualitative K Solubilization by PSB

K-solubilization of isolated strains was performed on agar medium amended by Mica as the only source of K. The nine isolates released K, among which, isolates BM11, CB13, CB19, and BT3S171 recorded the highest K release, followed by BT125, BM28, and BN313, while BM215 and BM218 were the lowest (Table 2).

### 3.5. Quantification of Organic Acids Produced by PSB Strains

As a result, the presence of eight recognized organic acids was demonstrated (Table 3). Acetic acid was vigorously generated by all strains, reaching concentrations of 144.76 mg/L in BM215, 102.98 mg/L in BM28, and 93.27 mg/L in BT125. The BSPs examined produced formic, propionic, isobutyric, butyric, isovaleric, caproic, and heptanoic acids, with considerable differences between all strains. Considering heptanoic acid is the least abundant, it was only found in BM11 and BT125 (Table 3). BM11 was the only strain producing all the organic acids tested.

### 3.6. Solubilization Test of the Three Forms of Phosphates

The PSB isolates were able to solubilize the three forms of inorganic P (Table 4, Table 5 and Table 6), The evaluation of the P solubilizing activity of the strain tested was carried out in NBRIP-broth medium every 48 h, for a period of 144 h. The phosphate solubilizing ability of the strain was increased up to 174.33 ± 12.5 μg/mL, 68.24 ± 6.53 μg/mL, and 84.15 ± 5.03 μg/mL for Ca_3_(PO_4_)_2_, AlPO_4_, and FePO_4_, respectively, as the pH decreased from 7.0 to 3.9, 3.1 and 3.3 for Ca_3_(PO_4_)_2_, AlPO_4_ and FePO_4_, respectively. 

**Figure 2:**
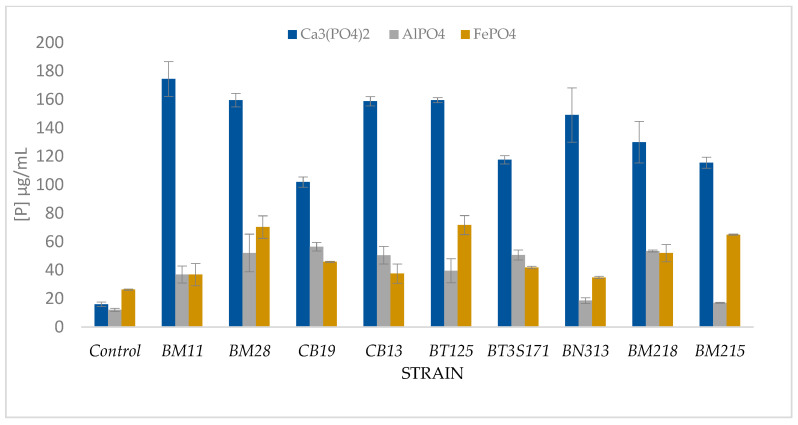
Solubilization of Ca_3_(PO_4_)_2_, AlPO_4_, and FePO_4_ by PSB after four days of incubation, each value is the mean of triplicates.

Maximal P solubilization was observed after 96 h of incubation for Ca_3_(PO_4_)_2_ and AlPO_4_, and after 144 h of incubation for FePO_4_ (Figure 2). Statistically, there was a significant difference (p ≤ 0.05) among the three treatments and PSB tested. There was a significant variation in the quantity of phosphorus liberated by the tested strains from the different inorganic phosphates tested. All of them were able to solubilize Ca_3_(PO_4_)_2_ more than FePO_4_ and AlPO_4_. Maximum Ca_3_(PO_4_)_2_ solubilization in NBRIP liquid medium was observed in BM11 (174.33 ± 12.5 μg/mL), followed by BT125 (159.48 ± 1.8 μg/mL), while for FePO_4_, the maximum solubilization was observed in BM28 (84.15 ± 5.03 μg/mL) followed by BT125 (77.44 ± 7.26 μg/mL), and for AlPO_4_, the maximum solubilization was observed in BT3S171 (68.24 ± 6.53 μg/mL) followed by CB13 (57.99 ± 11.83 μg/mL), and Modest P solubilization was observed in CB19 (112.51 ± 7.56 μg/mL), BM11 (33.59 ± 0.53 μg/mL), and BM215 (20.60 ± 1.22 μg/mL) for Ca_3_(PO_4_)_2_, FePO_4_, and AlPO_4_ successively. A decrease of pH was observed in all treatments. The results showed that the pH decreased after 48 h of incubation amongst all the strains where solubilization occurred. The greatest difference was found at the initial pH of 7.0, which decreased to 3.1 for strains inoculated in NBRIP broth medium amended with AlPO_4_.

## 4. Discussions

The objective of our study was to isolate PSB from phosphate solid sludge based on the NBRIP medium with Ca_3_(PO_4_)_2_ as the sole source of phosphorus, then to evaluate their ability to solubilize three forms of inorganic phosphates: Ca_3_(PO_4_)_2_, FePO_4_, and AlPO_4_. From a hundred isolates, we selected nine strains based on their ability of solubilization. Several authors have focused their studies on the solubilization of Ca_3_(PO_4_)_2_, FePO_4_, and AlPO_4_ [28,29,30,31]. The strains assessed in our investigation were able to solubilize Ca_3_(PO_4_)_2_; for FePO_4_ and AlPO_4_, the solubilization was minimal compared to Ca_3_(PO_4_)_2_, as well, the selected isolates showed high efficiency of solubilization for Ca_3_(PO_4_)_2_, since FePO_4_ and AlPO_4_ have a more complex structure than Ca_3_(PO_4_)_2_. Previous studies reported that the solubilization of Ca_3_(PO_4_)_2_ was the highest. Similar observations were reported by Reyes et al. [32] and Banik and Dey [28], when iron phosphate and hydroxyapatite were used. Correspondingly, it has been revealed by Devi and Thakuria [31], in their investigation on the PSB predominance in rice rhizospheric soils, that there was only 40.7% of 172 isolates dissolved aluminum phosphate (AlPO_4_). The soluble-P concentration ranged between 112.51 μg/mL and 174.33 μg/mL for Ca_3_(PO_4_)_2_, 84.15 μg/mL, 34.85 μg/mL for FePO_4_, 68.24 μg/mL, and 17.05 μg/mL for AlPO_4_. The isolate BM11 showed the highest potential to dissolve P from Ca_3_(PO_4_)_2_, and BT3S171 from AlPO_4_ and BM28 from FePO_4_. Additionally, in our study, the maximum solubilization of different phosphate sources was generally obtained after 96 h of incubation. On the 6th day, for some strains in all treatments, a decrease in phosphate solubilization had been observed. This decrease might be due to the diminution of nutrients in the medium [33]. 

Phosphate solubilization of the three forms of P followed by a decrease in pH of the medium was observed in the range 7.00–3.2. The decreasing pH of medium has likewise been reported from previous studies [34,35,36]. The production of organic acids by bacteria throughout their metabolic process induces the pH to decrease. Many PSB strains have been observed secreting a variety of organic acids, including acetic, citric, formic, oxalic, and formic acid, amongst many others. Organic acids [1,25,37,38,39,40,41] convert tricalcium phosphate to mono and dicalcium phosphate, allowing plants to receive phosphorus minerals. According to Nahas [42] and Anand et al. [43], organic acids generated by bacteria dissolve insoluble phosphate with a decrease in pH, chelation of cations, and interaction with phosphate on sorption sites in the soil. Thus, organic acids produced by PSB metabolism bound or chelate cations that bound P, therefore P solubility increases [44,45,46]. According to Mahidi [47] and Elfiati et al. [48], the molecular structure of organic acids, notably the number of carboxyl and hydroxyl groups, has a significant influence on their ability to chelate metal cations. The type and position of the ligand, in addition to the acid’s strength, determine its efficacy in the dissolving process.

In our investigation, PSB isolates produce organic acids in different quantities and types. The results of all isolated strains showed that acetic and isobutyric acid acids were the two major acids produced by all PSBs tested. PSB’s capacity to dissolve P is influenced by the amount and type of organic acids generated. All PSB isolates produced acetic, formic, and isobutyric acid, whereas eight isolates produced caproic acid, seven isolates produced isovaleric acid, six isolates produced propionic acid, and two isolates produced heptanoic acid. Organic acids have different abilities when it comes to releasing P bonds. Moreover, citric acid dissolved P more effectively than oxalic and malic acids, according to Hocking [49] and Hou et al. [50], and organic acids that can form a more stable complex with metal cations will be more successful in releasing aluminum and iron from soil minerals, allowing for more phosphorus to be released. In our study, four genera were determined *Pseudomonas*, *Serratia*, *Pantoea*, and *Enterobacter*, PSBs are diversified in nature [51], according to Biswas et al. [52], Sulbaran et al. [53], Bendjelloul et al. [54], and Liu et al. [36]; bacteria belonging to the genera *Pseudomonas*, *Enterobacter*, *Serratia*, and *Pantoea* are potent PSMs. Moreover, *Pseudomonas* genera are among the most efficient solubilizers of inorganic phosphate [55]. 

The strains investigated were assessed for PGP characteristics (IAA, siderophores, and HCN) and potassium solubilization. In addition to their ability to solubilize inorganic phosphates, PSB efficiency is due not only to their potential to raise P availability, but also to their capacity to produce growth-regulating agents such as IAA, a growth regulator that aids in cell growth and division, stress resistance, root lengthening, nitrogen fixation stimulation, and biosynthesis of various metabolites [56]. All PSB isolates generate IAA, with different quantities between isolates. Likewise, in the secretion of siderophores, which plays a very important role in the release of iron [57], siderophores behave as dissolving agents for iron from minerals or organic compounds under conditions of iron restriction. As well, siderophores can form stable complexes with additional metals that are environmentally damaging [58]. In our study, all PSB isolates produced siderophores. On the other hand, plants require K as the third most important macronutrient. More than three-quarters of the K in agricultural soils are in the form of insoluble organic and inorganic molecules or complexes, which are inaccessible to plants [59], such as BSP strains, where the use of K-solubilizing bacteria as a biofertilizer might be an environmentally friendly alternative technique for plant K uptake. BSPs have the potential to be a useful biofertilizer. Phosphate-solubilizing, potassium-solubilizing, and significant growth-promotion effects on plant development have been observed for many BSP species. In the present study, we have clearly demonstrated that the isolated PSB can be a potential plant microbial agent that could be used to promote plant growth even in acidic or calcareous soil.

## 5. Conclusions

To conclude, nine strains were isolated in this study; they belong to the genus *Pseudomonas*, *Serratia*, *Pantoea*, and *Enterobacter*. The PSB isolates were able to solubilize the three forms of P: Ca_3_(PO_4_)_2_ > FePO_4_ > AlPO_4_. Additionally, the strains showed plant growth-promoting traits, thereby indicating that they have a role in enhancing plant growth. It is expected that the use of P-solubilizers will considerably minimize the environmental impact of chemical fertilizer use. Thus, the ability of the strains studied to solubilize the three forms of inorganic phosphate tested in our study, in addition to the PGP traits and the ability of K-solubilization, may make them good candidates as biofertilizers. Due to this, more research should be performed to see if yields can be grown with them as biofertilizers in greenhouses and on the ground, which may facilitate long-term P management in sustainable agriculture.

## Figures and Tables

**Table 1:** Phosphate solubilization index (PSI) of the selected PSB strains.

Strains	Colony Diameter (cm)	Halo Zone Diameter (cm)	PSI = C + H/C
BM11	0.98	2.3	3.33 ^b^
BM28	0.78	2.6	4.10 ^a^
CB19	0.58	2.4	4.79 ^d^
CB13	0.70	1.5	2.77 ^c^
BT125	0.68	2.3	4.12 ^a^
BT3S171	0.65	1.4	2.77 ^c^
BN313	0.63	1.6	3.11 ^b^
BM218	0.95	2.9	4.00 ^a^
BM215	0.78	2.1	3.42 ^b^

Values superscripted by different letters in the same column are significantly different (*p* ≤ 0.05).

**Table 2:** Morphological, biochemical characterization, PGP traits, and K solubilization of PSB strains.

		Strain								
		BM11	BM28	CB19	CB13	BT125	BT3S171	BN313	BM218	BM215
Colony color		Red	Yellowish	Yellowish	Yellow-ish	Yellow-ish	Yellowish	Yellow-ish	Yellowish	Yellowish
Gram		−	−	−	−	−	−	−	−	−
β−galactosidase		+	+	+	+	−	+	−	+	+
Arginine dihydrolase		+	+	−	−	−	−	+	−	+
Lysine decarboxylase		−	−	−	−	−	−	−	−	−
Ornithine decarboxylase		−	+	−	−	−	−	+	−	+
Citrate utilization		+	+	−	−	−	−	−	−	+
H_2_S production		−	−	−	−	−	−	−	−	−
Urease		−	−	−	−	+	−	−	−	−
Tryptophan deaminase		−	−	−	−	−	−	−	−	−
Indole production		−	−	−	−	−	−	−	−	−
Acetoin production		−	−	+	−	−	−	−	−	−
Gelatinase		+	+	+	+	+	−	+	+	+
Sugar fermentation	Glucose	+	+	+	+	−	+	+	+	+
	Mannitol	+	+	−	+	−	−	−	+	+
	Inositol	−	−	−	−	−	−	−	−	−
	Sorbitol	−	−	+	−	−	−	−	−	−
	Rhamnose	−	+	+	+	−	−	−	−	+
	Sucrose	+	−	+	+	−	−	−	+	−
	Melibiose	+	−	+	−	−	−	+	+	−
	Amygdalin	+	+	+	+	−	−	−	+	+
	Arabinose	+	+	+	+	−	−	+	+	+
PGP traits	Indole-3-acetic acid production (µg/mL)	2.6 ± 0.0 ^a^	7.7 ± 0.03 ^b^	3.2 ± 0.03 ^a^	8.7 ± 0.1 ^c^	3.9 ± 0.4 ^a^	43.8 ± 0.39 ^d^	1.3 ± 0.09 ^e^	20.5 ± 0.03 ^f^	15.9 ± 0.2 ^g^
	Siderophore	+	+	+	+	++	++	++	++	++
	Hydrogen cyanide	−	−	−	−	++	−	−	−	−
K solubilization		+++	++	+++	+++	++	+++	++	+	+

(+): Positive reaction; (−): Negative reaction− = Negative; + = Moderate; ++ = High; +++ = Very high. Values superscripted by different letters in the same line are significantly different (*p* ≤ 0.05).

**Table 3:** Quantification of organic acids produced by PSB strains in liquid NBRIP culture medium with Ca_3_(PO_4_)_2_ as sole source of P.

Strain	Acetic	Formic	Propionic	Isobutyric	Butyric	Isovaleric	Caproic	Heptanoic
	mg/L							
BM11	40.62	3.99	1.59	6.86	8.53	1.32	0.59	0.51
BM28	102.98	1.78	9.14	5.39	ND	0.10	2.19	ND
CB19	28.67	3.42	ND	3.89	2.95	1.21	7.29	ND
CB13	27.01	2.93	ND	4.23	2.88	0.31	5.60	ND
BT125	93.27	3.94	0.38	5.48	ND	0.98	7.87	0.20
BT3S171	73.52	3.70	0.79	8.12	ND	ND	1.64	ND
BN313	60.94	7.38	5.80	4.66	3.89	ND	ND	ND
BM218	28.77	2.80	ND	4.55	2.37	0.90	4.58	ND
BM215	144.76	2.71	8.67	8.01	ND	0.82	1.34	ND

ND: not detected.

**Table 4:** Quantitative estimation of P solubilization in NBRIP broth medium with Ca_3_(PO_4_)_2_ as sole source of phosphorus by selected PSB.

Strain	Hour of Incubation							
	0		48		96		144	
	[P] µg/mL	pH	[P] µg/mL	pH	[P] µg/mL	pH	[P] µg/mL	pH
**Control**	12.95 ± 1.39 ^a^	7.0 ± 0.2 ^a^	16.61 ± 2.62 ^a^	6.3 ± 0.04 ^a^	16.00 ± 1.57 ^a^	6.3 ± 0.00 ^a^	12.67 ± 1.46 ^a^	6.51 ± 0.0 ^a^
**BM11**	20.65 ± 0.09 ^a^	7.0 ± 0.2 ^a^	109.07 ± 5.4 ^d^	4.2 ± 0.02 ^b^	174.33 ± 12.19 ^e^	4.1 ± 0.08 ^d^	101.30 ± 6.49 ^b^	4.0 ± 0.25 ^ef^
**BM28**	14.85 ± 0.09 ^a^	7.0 ± 0.2 ^a^	106.83 ± 1.23 ^d^	4.7 ± 0.06 ^c^	159.48 ± 4.73 ^de^	4.4 ± 0.1 ^bc^	111.15 ± 8.79 ^bc^	4.6 ±0.2 ^ef^
**CB19**	13.23 ± 0.73 ^a^	7.0 ± 0.2 ^a^	81.10 ± 35.36 ^b^	4.1 ± 0.08 ^b^	101.90 ± 3.55 ^b^	4.2 ± 0.7 ^cd^	112.51 ± 7.56 ^bc^	4.6 ±0.2 ^cd^
**CB13**	17.47 ± 0.07 ^a^	7.0 ± 0.2 ^a^	110.34 ± 5.74 ^d^	4.2 ± 0.05 ^b^	158.72 ± 3.27 ^de^	4.1 ± 0.97 ^d^	107.21 ± 10.7 ^bc^	4.3 ± 0.3 ^de^
**BT125**	15.44 ± 0.30 ^a^	7.0 ± 0.2 ^a^	107.90 ± 5.60 ^d^	4.2 ± 0.04 ^b^	159.48 ± 1.83 ^de^	4.1 ± 0.00 ^d^	117.36 ± 0.78 ^c^	4.1 ± 0.0 ^bcd^
**BT3S171**	15.58 ± 2.03 ^a^	7.0 ± 0.2 ^a^	23.87 ± 3.38 ^a^	5.9 ± 0.09 ^e^	117.51 ± 2.92 ^bc^	4.0 ± 0.27 ^d^	111.45 ± 6.14 ^bc^	3.9 ± 0.0 ^b^
**BN313**	20.29 ± 0.10 ^a^	7.0 ± 0.2 ^a^	88.72 ± 28.6 ^bc^	4.9 ± 0.28 ^d^	149.03 ± 19.06	4.5 ± 0.22 ^b^	114.18 ± 2.53 ^c^	4.3 ± 0.25 ^cde^
**BM218**	12.03 ± 0.09 ^a^	7.0 ± 0.2 ^a^	104.4 ± 2.45 ^cd^	4.2 ± 0.05 ^b^	129.93 ± 14.54 ^c^	4.1 ± 0.05 ^d^	106.00 ± 8.19 ^bc^	4.06 ± 0.2 ^bc^
**BM215**	14.21 ± 1.15 ^a^	7.0 ± 0.2 ^a^	62.06 ± 6.95 ^b^	4.6 ± 0.03 ^c^	115.54 ± 3.88 ^bc^	4.5 ± 0.0 ^b^	112.96 ± 4.59 ^bc^	4.7 ±0.2 ^f^

Values are the mean of *n* = 3, expressed with the standard error of means. Values superscripted by different letters are significantly different (*p* ≤ 0.05).

**Table 5:** Quantitative estimation of P solubilization in NBRIP broth medium with AlPO_4_ as sole source of phosphorus by selected PSB.

Strain	Hour of Incubation							
	0		48		96		144	
	**[P] µg/mL**	pH	**[P] µg/mL**	pH	**[P] µg/mL**	pH	**[P] µg/mL**	pH
**Control**	24.42 ± 1.11 ^a^	7.0 ± 0.2 ^a^	27.81 ± 0.37 ^a^	6.9 ± 0.17 ^a^	26.29 ± 0.39 ^a^	5.8 ± 0.2 ^a^	29.44 ± 0.44 ^a^	5.1 ± 0.1 ^a^
**BM11**	38.18 ± 3.43 ^b^	7.0 ± 0.2 ^a^	32.83 ± 0.78 ^a^	3.9 ± 0.0 ^c^	36.90 ± 7.82 ^bc^	3.8 ± 0.0 ^c^	33.59 ± 0.53 ^a^	3.8 ± 0.0 ^c^
**BM28**	33.48 ± 2.74 ^b^	7.0 ± 0.2 ^a^	57.84 ± 2.07 ^cde^	4.1 ± 0.0 ^d^	70.26 ± 7.92 ^e^	4.0 ± 0.0 ^cd^	84.15 ± 5.03 ^e^	3.7 ± 0.0 ^cd^
**CB19**	41.90 ± 2.54 ^b^	7.0 ± 0.2 ^a^	51.30 ± 0.42 ^cd^	3.3 ± 0.0 ^b^	45.81 ± 0.37 ^cb^	3.3 ±0.0 ^b^	48.43 ± 1.85 ^df^	3.3 ±0.0 ^b^
**CB13**	31.21 ± 1.56 ^b^	7.0 ± 0.2 ^a^	61.39 ± 3.3 ^e^	4.1 ± 0.0 ^d^	37.52 ± 6.74 ^cd^	3.3 ± 0.2 ^b^	36.50 ± 0.82 ^a^	3.3 ± 0.12 ^b^
**BT125**	36.79 ± 5.17 ^b^	7.0 ± 0.2 ^a^	40.8 ± 1.1 ^b^	3.7 ± 0.2 ^b^	71.64 ± 6.72 ^e^	4.0 ± 0.1 ^cd^	77.44 ±7.26 ^e^	4.0 ± 0.78 ^d^
**BT3S171**	34.38 ± 1.38 ^b^	7.0 ± 0.2 ^a^	61.39 ± 3.3 ^cde^	3.4 ± 0.0 ^b^	41.85 ± 0.93 ^bc^	3.4 ± 0.0 ^b^	35.60 ±2.54 ^a^	3.4 ± 0.0 ^b^
**BN313**	39.76 ± 0.04 ^b^	7.0 ± 0.2 ^a^	40.05 ± 2.3 ^b^	4.2 ± 0.0 ^d^	34.85 ± 0.79 ^ab^	4.2 ± 0.0 ^d^	15.86 ± 4.36 ^b^	4.0 ± 0.0 ^d^
**BM218**	47.76 ± 1.54 ^b^	7.0 ± 0.2 ^a^	58.18 ± 6.8 ^de^	3.4 ± 0.0 ^b^	51.96 ± 6.09 ^d^	3.4 ± 0.0 ^b^	44.86 ± 3.83 ^d^	3.3 ± 0.0 ^b^
**BM215**	33.14 ± 0.71 ^b^	7.0 ± 0.2 ^a^	50.96 ± 5.61 ^c^	3.9 ± 0.0 ^c^	64.92 ± 0.43 ^e^	4.2 ± 0.0 ^d^	51.87 ± 3.58 ^f^	3.8 ± 0.0 ^d^

Values are the mean of *n* = 3, expressed with the standard error of means. Values superscripted by different letters are significantly different (*p* ≤ 0.05).

**Table 6:** Quantitative estimation of P solubilization in NBRIP broth medium with FePO_4_ as sole source of phosphorus by selected PSB.

Strain	Hour of Incubation							
	0		48		96		144	
	**[P] µg/mL**	pH	**[P] µg/mL**	pH	**[P] µg/mL**	pH	**[P] µg/mL**	pH
**Control**	24.42 ± 1.11 ^a^	7.0 ± 0.2 ^a^	27.81 ± 0.37 ^a^	6.9 ± 0.17 ^a^	26.29 ± 0.39 ^a^	5.8 ± 0.2 ^a^	29.44 ± 0.44 ^a^	5.1 ± 0.1 ^a^
**BM11**	38.18 ± 3.43 ^b^	7.0 ± 0.2 ^a^	32.83 ± 0.78 ^a^	3.9 ± 0.0 ^c^	36.90 ± 7.82 ^bc^	3.8 ± 0.0 ^c^	33.59 ± 0.53 ^a^	3.8 ± 0.0 ^c^
**BM28**	33.48 ± 2.74 ^b^	7.0 ± 0.2 ^a^	57.84 ± 2.07 ^cde^	4.1 ± 0.0 ^d^	70.26 ± 7.92 ^e^	4.0 ± 0.0 ^cd^	84.15 ± 5.03 ^e^	3.7 ± 0.0 ^cd^
**CB19**	41.90 ± 2.54 ^b^	7.0 ± 0.2 ^a^	51.30 ± 0.42 ^cd^	3.3 ± 0.0 ^b^	45.81 ± 0.37 ^cb^	3.3 ±0.0 ^b^	48.43 ± 1.85 ^df^	3.3 ±0.0 ^b^
**CB13**	31.21 ± 1.56 ^b^	7.0 ± 0.2 ^a^	61.39 ± 3.3 ^e^	4.1 ± 0.0 ^d^	37.52 ± 6.74 ^cd^	3.3 ± 0.2 ^b^	36.50 ± 0.82 ^a^	3.3 ± 0.12 ^b^
**BT125**	36.79 ± 5.17 ^b^	7.0 ± 0.2 ^a^	40.8 ± 1.1 ^b^	3.7 ± 0.2 ^b^	71.64 ± 6.72 ^e^	4.0 ± 0.1 ^cd^	77.44 ±7.26 ^e^	4.0 ± 0.78 ^d^
**BT3S171**	34.38 ± 1.38 ^b^	7.0 ± 0.2 ^a^	61.39 ± 3.3 ^cde^	3.4 ± 0.0 ^b^	41.85 ± 0.93 ^bc^	3.4 ± 0.0 ^b^	35.60 ±2.54 ^a^	3.4 ± 0.0 ^b^
**BN313**	39.76 ± 0.04 ^b^	7.0 ± 0.2 ^a^	40.05 ± 2.3 ^b^	4.2 ± 0.0 ^d^	34.85 ± 0.79 ^ab^	4.2 ± 0.0 ^d^	15.86 ± 4.36 ^b^	4.0 ± 0.0 ^d^
**BM218**	47.76 ± 1.54 ^b^	7.0 ± 0.2 ^a^	58.18 ± 6.8 ^de^	3.4 ± 0.0 ^b^	51.96 ± 6.09 ^d^	3.4 ± 0.0 ^b^	44.86 ± 3.83 ^d^	3.3 ± 0.0 ^b^
**BM215**	33.14 ± 0.71 ^b^	7.0 ± 0.2 ^a^	50.96 ± 5.61 ^c^	3.9 ± 0.0 ^c^	64.92 ± 0.43 ^e^	4.2 ± 0.0 ^d^	51.87 ± 3.58 ^f^	3.8 ± 0.0 ^d^

Values are the mean of *n* = 3, expressed with the standard error of means. Values superscripted by different letters are significantly different (*p* ≤ 0.05).

## Data Availability

The data presented in the study are available in article.
